# Utilization Trends of Phosphodiesterase Type-5 Inhibitors for Erectile Dysfunction Between 2019 and 2023 in Tanzania

**DOI:** 10.7759/cureus.58419

**Published:** 2024-04-16

**Authors:** Raphael Z Sangeda, Auleria W Kadinde, Cassian F Masatu, Yonah Hebron Mwalwisi, Khadija I Yahya-Malima, Adam M Fimbo

**Affiliations:** 1 Pharmaceutical Microbiology, Muhimbili University of Health and Allied Sciences, Dar es Salaam, TZA; 2 Medicines Control, Tanzania Medicines and Medical Devices Authority, Dodoma, TZA; 3 Nursing Management, Muhimbili University of Health and Allied Sciences, Dar es Salaam, TZA

**Keywords:** drug utilization research, atc classification, ddd, daily defined doses, pde5is, phosphodiesterase 5 inhibitors, tanzania, sildenafil, viagra, erectile dysfunction

## Abstract

Introduction

Erectile dysfunction (ED) profoundly affects millions of people globally, including interfering with mental health and quality of life. Phosphodiesterase type-5 inhibitors (PDE5Is) such as sildenafil are pivotal in ED treatment. This study aimed to examine the utilization patterns of PDE5Is in Tanzania.

Materials and methods

In this retrospective longitudinal study, data on sildenafil and other similar PDE5Is imported between 2019 and 2023 were sourced from the Tanzania Medicines and Medical Devices Authority (TMDA). Pre-processing and visualization were performed using Microsoft Power BI Desktop, and further analysis was performed using IBM SPSS Statistics for Windows, Version 26 (Released 2019; IBM Corp., Armonk, New York, United States). Utilization trends were ascertained through curve fitting, Holt's linear trend model, and autoregressive integrated moving average (ARIMA) models. The defined daily doses (DDDS) per 1000 inhabitants (DID) were calculated using the World Health Organization (WHO) Anatomical Therapeutic Chemical (ATC) Classification System and the DDD methodology endorsed by the WHO Collaborating Centre for Drug Statistics Methodology.

Results

Between 2019 and 2023, there was a pronounced increase in the importation of approximately 587 consignments of PDE5Is. Employing the Holt model (R-square = 0.843), a substantial increase from 0.220910 DID in 2019 to 0.534272 DID by 2025 was observed and anticipated. The period witnessed sildenafil dominating 75.5% of the total use, with Erecto being the most consumed brand (37.6% of total DID). Notably, 2022 had the highest surge (27.2% of the total), albeit a slight decline was observed in 2023 (20.5%). This trend was supported by a linear regression model (R-square = 0.889).

Conclusion

We found increasing annual trends of PDE5Is of utilization. This requires critical oversight and effective policies to ensure appropriate use and risk minimization.

## Introduction

Erectile dysfunction (ED), characterized by the inability to achieve or sustain an erection sufficient for satisfactory sexual performance, is a medical condition affecting an estimated 322 million individuals worldwide, including 30 million people in Africa [1,2]. Approximately 30 to 50 million men in the United States and 150 million people globally grapple with sexual dysfunction owing to their inability to maintain erection. This problem is widespread among older individuals, with about half of men over the age of 40 years experiencing dysfunction [3].

ED, also known as impotence, has severe social implications for the affected individuals and their communities. This prevalent sexual dysfunction in men often arises from inadequate blood flow to the penis owing to various organic, relational, or psychological factors [2]. These include old age, stress, mental health disorders, prostate surgery, substance abuse, diabetes, multiple sclerosis, smoking, or excessive cycling [2,3].

ED can significantly undermine a man's quality of life and most patients experience depression and anxiety associated with sexual performance. These issues can adversely impact partners' sexual satisfaction and couples’ overall quality of life. Increased frequency of sildenafil use has been linked to decreased confidence in performance, which negatively affects erectile function. It has been reported that men who use ED medications recreationally face an increased risk of psychological dependency, which potentially leads to psychological symptoms [4,5]. Given the severity and consequences of ED, the search for effective remedies is ongoing.

Sildenafil, commonly known as Viagra, is a phosphodiesterase type-5 inhibitor (PDE5I) that works by relaxing the smooth muscle lining of blood vessels, thereby causing dilatation and enhancing blood flow [2,6,7]. This mechanism underpinned the intended use of sildenafil as an antihypertensive medicine, which was later modified to induce erection [7]. The use of sildenafil is associated with adverse effects ranging from less severe symptoms, such as facial flushing, headache, nasal congestion, dyspepsia, dizziness, nightmares, palpitations, lethargy, and muscle pain, to more severe symptoms, such as acute myocardial infarction [8]. Consequently, the drug may not be suitable for individuals with cardiovascular diseases or asymptomatic patients because the same may exacerbate these conditions [8,9]. Tadalafil, vardenafil, and avanafil are other PDE5Is commonly used for ED.

Despite the recognized importance of understanding sildenafil and other PDE5Is utilization trends and their implications, the full extent of this conundrum in Tanzania remains largely undefined. Acknowledging the crucial need for detailed insights into the utilization of sildenafil and other PDE5Is in Tanzania, this study was conducted to address the existing knowledge gap by leveraging importation data from the Tanzania Medicines and Medical Devices Authority (TMDA). Focusing on a comprehensive evaluation of sildenafil and PDE5Is importation over five years, this study seeks to uncover the patterns and rationality behind their consumption. The survey findings will enable the TMDA, as the regulatory authority in Tanzania, to construe importation dynamics, devise measures to control the use of sildenafil and other PDE5Is, and safeguard public health.

## Materials and methods

Study design

This study utilized a retrospective, longitudinal, analytical design to analyze PDE5Is utilization trends, drawing importation data on human medicines from January 1, 2019, to December 31, 2023, within mainland Tanzania.

Study setting

The study was conducted in the United Republic of Tanzania, which is geographically situated at 6.3690 °S, 34.8888 °E. The country borders eight neighboring countries, and the Indian Ocean is in the eastern part. The country has numerous entry points for pharmaceuticals imported through seaports and airports, including Dar es Salaam Airport and Seaport, Kilimanjaro Airport, and several other national terrestrial border checkpoints such as Sirari, Horohoro, Namanga, Tunduma, and Mutukula.

Data source

The data for this study were sourced from the TMDA database and included information on sildenafil and PDE5Is imported for systemic use in mainland Tanzania. The data captured details regarding the port of entry at air, sea, and border checkpoints. All importers of medicines in Tanzania are required to request permits from TMDA. Importation data was, thus, used to estimate the utilization trend of PDE5Is under the assumption that all imported medicines are utilized within Tanzania.

Exclusion criteria

All records that lacked permit numbers, reference numbers, or permit issue dates were excluded because of their inability to verify their importation year. Records outside the scope of the study timeframe were omitted.

Data collection

Regarding data collection, TMDA, functioning as the National Medicines Regulatory Authority (NMRA), regulates the importation of medicines into mainland Tanzania and, therefore, mandates that importers apply for import permits. Once the application is evaluated and approved, permits are issued and stored in TMDA's Regulatory Information Management System (RIMS). The extracted records included the importation date, generic and brand names of medicines, strength, quantity, company (supplier or importer), price, currency, product manufacturer, and country of origin.

The defined daily dose (DDD) per 1000 inhabitants per year (DID) was calculated by determining the total amount of medications in milligrams for each medicine and then dividing it by the DDD for the generic name of the medicine to obtain the total DDDs. The total DDD was then divided by the population size, standardized to represent the DDD per 1000 inhabitants, and adjusted to reflect annual utilization by dividing it by the number of days in a year (365), consistent with the DDD list available on the website of the World Health Organization (WHO) Collaborating Center for Drug Statistics Methodology [10].

$\text{DID} = \left( \frac{\text{Total drug amount in mg}}{\text{DDD for the generic drug}} \times \frac{1000}{\text{population}} \right) \div 365$

Data analysis

This involved merging, pivoting, and aggregating the data files using Microsoft Excel 2016. The strength, pack size, and quantity of sildenafil were converted into milligrams, grams, and kilograms, respectively, to quantify its utilization. Visualizations, such as tables and graphs, demonstrated sildenafil utilization trends. Data were cleaned, aggregated, and visualized using Microsoft Power BI Software and then entered into the IBM SPSS Statistics for Windows, Version 26 (Released 2019; IBM Corp., Armonk, New York, United States) for time-series and regression analyses to determine the annual trend of PDE5Is utilization [11,12].

Time series was achieved through advanced modeling techniques, including autoregressive integrated moving average (ARIMA), where Holt linear trend model incorporated via the Expert Modeler feature with options for automatic ARIMA and exponential smoothing (EXSMOOTH) selection, along with seasonal adjustments, to examine the linear trends and seasonal variations of the data. The value for 2023 was omitted from the curve fitting and forecasting processes because of its outlier status, with a recorded value of 0.29687 DIDs, which deviates from the expected trends.

## Results

A total of 587 importation records were collected from 2019 to 2023, with the highest level of consignment imported in 2022 (35.1%) followed by 29.3% (2023) (Table 1).

**Table 1:** Number of importation permits and proportions in annual contribution

Year	Number of consignments	Proportions
2019	59	10.1%
2020	66	11.2%
2021	84	14.3%
2022	206	35.1%
2023	172	29.3%
Total	587	100%

**Figure 1:**
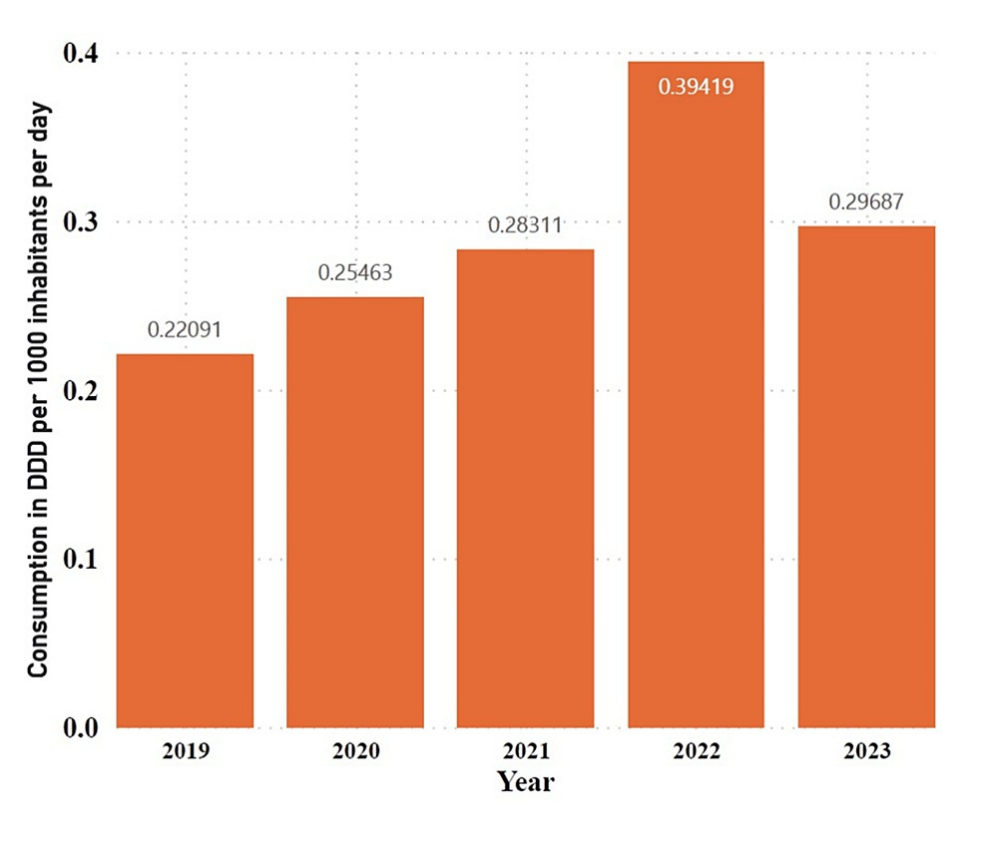
Annual defined daily doses per 1000 inhabitants per day contributions to the utilization of phosphodiesterase type-5 inhibitors DDD: Defined daily dose

Considering the DID, the five-year total was 1.44970 DIDs. There was a general upward trend in utilization from 2019 to 2023, with a notable increase in 2022 to 0.39419 DID (27.2%) of the cumulative total, followed by a slight decrease in 2023 to 0.29687 DID (20.5%) of the cumulative total (Figure 1).

**Figure 2:**
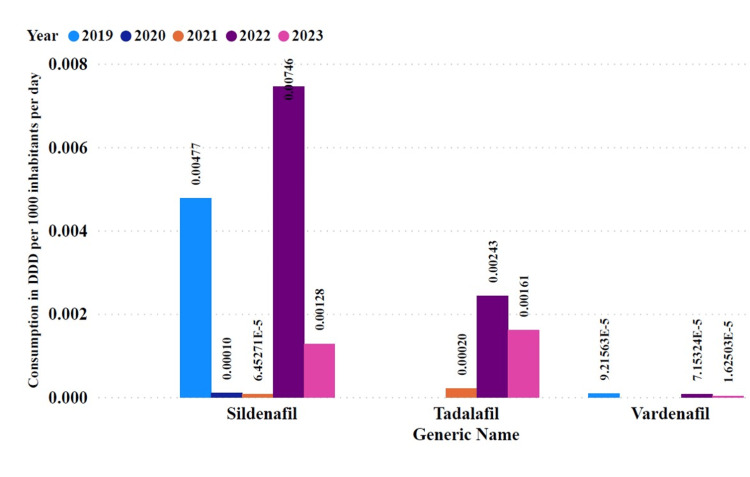
Annual utilization of phosphodiesterase type-5 inhibitors expressed in defined daily doses per 1000 inhabitants per day by generic name DDD: Defined daily dose

Among the PDE5Is, sildenafil displayed the highest utilization rates over the five years studied. It accounted for 75.5% of all PDE5Is utilization, compared to tadalafil and vardenafil, which accounted for 23.45% and 0.989%, respectively. Annual utilization of DID decreased in 2020 and 2021 compared to 2019, increased in 2022, and decreased again in 2023 (Figure 2).

**Figure 3:**
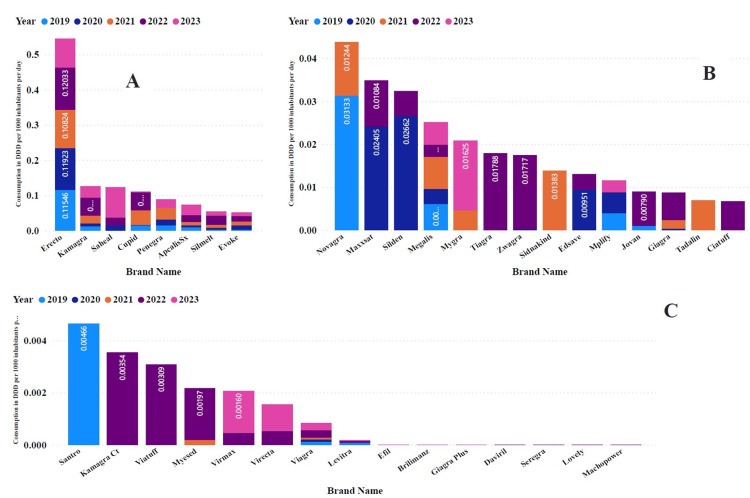
DID contribution of each brand of PDE5Is utilization in Tanzania. The top eight products are in panel A with DID > 0.05, panel B with DID between 0.05 and 0.005, and panel C with DID < 0.005 DID: Defined daily doses per 1000 inhabitants per day; PDE5Is: Phosphodiesterase type-5 inhibitors; DDD: Defined daily dose

A total of 37 diverse brands of PDE5Is were consumed during the study period. Upon closer examination of the various brands of PDE5Is, Erecto emerged as the most popular choice in Tanzania, with a remarkable total DID of 0.54451 (37.6%) over five years, cementing its position as the dominant brand among PDE5Is (Figure 3).

The cumulative DID totals for Erecto and the other 12 top brands, Kamagra, Saheal, Cupid, Penegra, ApcalisSx, Silmelt, Evoke, Novagra, Maxxsat, Silden, Megalis, and Mygra, accounted for 90% of all PDE5Is imported into the country. Of particular note is the fact that Viagra had a DID of 1.44868 (0.1%) (Table 2).

**Table 2:** Cumulative DID and percentage contribution of each brand to PDE5Is utilization in Tanzania. The top 12 products constituted 90% of all imported products over the five-year period NA: No import in that year; "-": Utilization DID value <0. 00001; DID: Defined daily doses per 1000 inhabitants; PDE5Is: Phosphodiesterase type-5 inhibitors

	Year							
Brand name	2019	2020	2021	2022	2023	Brand Total	Cumulative total	Cumulative %
Erecto	0.11546	0.11923	0.10824	0.12033	0.08125	0.54451	-	-
Kamagra	0.01205	0.00916	0.02234	0.05064	0.03194	0.12613	0.54451	37.6
Saheal	NA	0.01483	NA	0.02325	0.0845	0.12258	0.67064	46.3
Cupid	0.01551	0.00143	0.0419	0.05087	0.0013	0.11101	0.79322	54.7
Penegra	0.01569	0.01597	0.03319	NA	0.02328	0.08813	0.90423	62.4
ApcalisSx	0.00949	0.00627	0.00973	0.01931	0.02841	0.07321	0.99236	68.5
Silmelt	0.00311	0.00594	0.00817	0.02562	0.01072	0.05356	1.06557	73.5
Evoke	0.00246	0.01248	0.01197	0.01531	0.00845	0.05067	1.11913	77.2
Novagra	0.03133	NA	0.01244	NA	NA	0.04377	1.1698	80.7
Maxxsat	NA	0.02405	NA	0.01084	NA	0.03489	1.21357	83.7
Silden	NA	0.02662	NA	0.00579	NA	0.03241	1.24846	86.1
Megalis	0.00605	0.00362	0.00736	0.00288	0.00517	0.02508	1.28087	88.4
Mygra	NA	NA	0.00461	NA	0.01625	0.02086	1.30595	90.1
Tiagra	NA	NA	NA	0.01788	NA	0.01788	1.32681	91.5
Zwagra	NA	0.00021	NA	0.01717	NA	0.01738	1.34469	92.8
Sidnakind	NA	NA	0.01383	NA	NA	0.01383	1.36207	94.0
Edsave	NA	0.00951	NA	0.00348	NA	0.01299	1.3759	94.9
Mplify	0.00392	0.00494	NA	NA	0.00268	0.01154	1.38889	95.8
Jovan	0.00099	NA	NA	0.0079	NA	0.00889	1.40043	96.6
Giagra	NA	0.00027	0.00214	0.0063	NA	0.00871	1.40932	97.2
Tadalin	NA	NA	0.00691	NA	NA	0.00691	1.41803	97.8
Ciatuff	NA	NA	NA	0.00666	NA	0.00666	1.42494	98.3
Santro	0.00466	NA	NA	NA	NA	0.00466	1.4316	98.8
Kamagra Ct	NA	NA	NA	0.00354	NA	0.00354	1.43626	99.1
Viatuff	NA	NA	NA	0.00309	NA	0.00309	1.4398	99.3
Myesed	NA	NA	0.0002	0.00197	NA	0.00217	1.44289	99.5
Virmax	NA	NA	NA	0.00046	0.0016	0.00206	1.44506	99.7
Virecta	NA	NA	NA	0.00054	0.00102	0.00156	1.44712	99.8
Viagra	0.00011	0.0001	0.00006	0.00029	0.00026	0.00082	1.44868	99.9
Levitra	0.00009	NA	NA	0.00007	0.00001	0.00017	1.4495	100.0
Efil	NA	NA	NA	NA	0.00001	0.00001	1.44967	100.0
Lovely	NA	NA	NA	-	NA	-	1.44968	100.0
Daviril	NA	NA	NA	-	NA	-	1.44968	100.0
Seregra	NA	NA	NA	-	NA	-	1.44968	100.0
Brilimanz	NA	NA	NA	NA	-	-	1.44968	100.0
Machopower	NA	NA	NA	-	NA	-	1.44968	100.0
Giagra Plus	NA	NA	NA	NA	-	-	1.44968	100.0
Year total	0.22092	0.25463	0.28309	0.39419	0.29685	1.44968	1.44968	100.0

**Figure 4:**
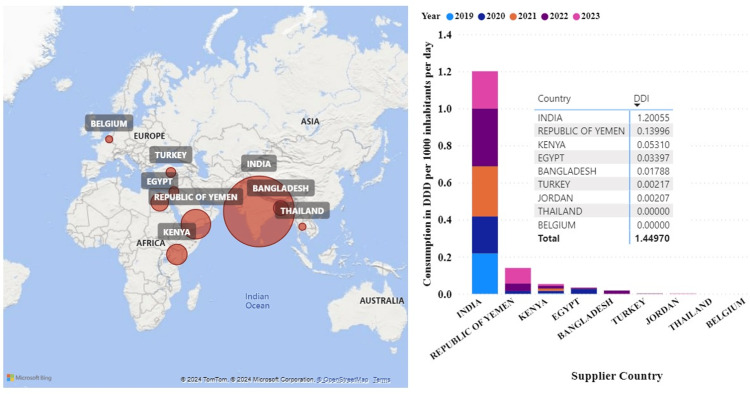
Top importers of phosphodiesterase type-5 inhibitors by country. The bubble size in panel A corresponds to the DID of medicines imported into Tanzania from that country. Panel B shows the annual size of the DIDs consumed in Tanzania by the respective countries The map was created using Microsoft Power BI Desktop software and geographic data from OpenStreetMap contributors licensed under the Open Data Commons Open Database License (ODbL). DID: Defined daily doses per 1000 inhabitants per day; DDD: Defined daily dose

Based on the data analyzed, India was the top exporter of PDE5Is to Tanzania. Between 2019 and 2023, India supplied 80% of all imports. Other countries included Kenya, the Republic of Yemen, and Egypt (Figure 4).

**Figure 5:**
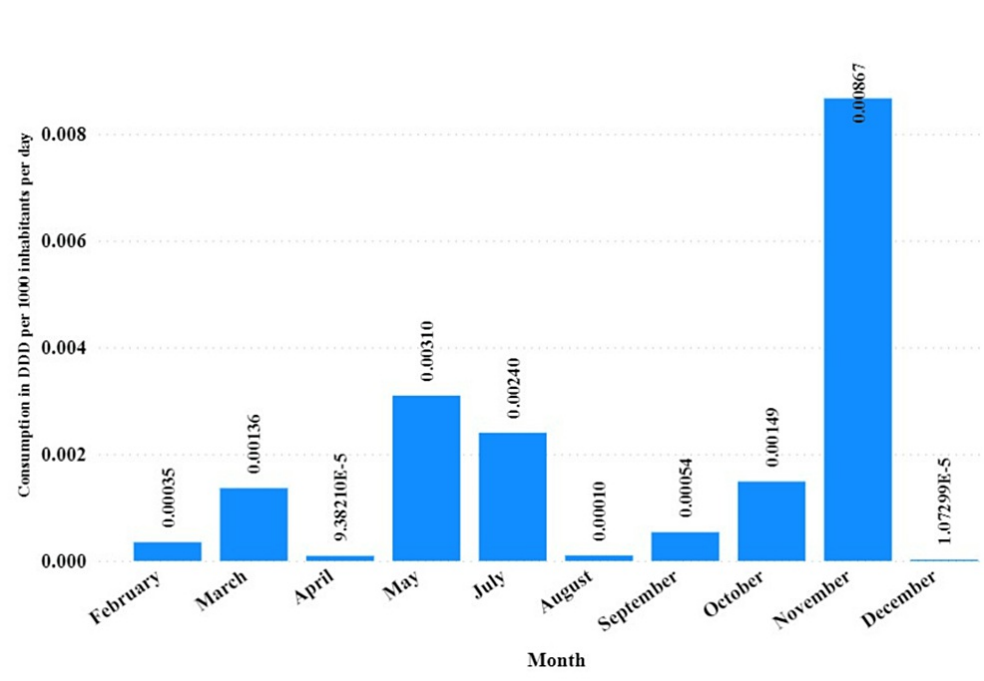
Monthly utilization trends of phosphodiesterase type-5 inhibitors from January 2019 to December 2023 DDD: Defined daily dose

Overall, in the five years investigated, PDE5Is had the highest utilization trends in November compared to other months, followed by May and July (Figure 5).

**Figure 6:**
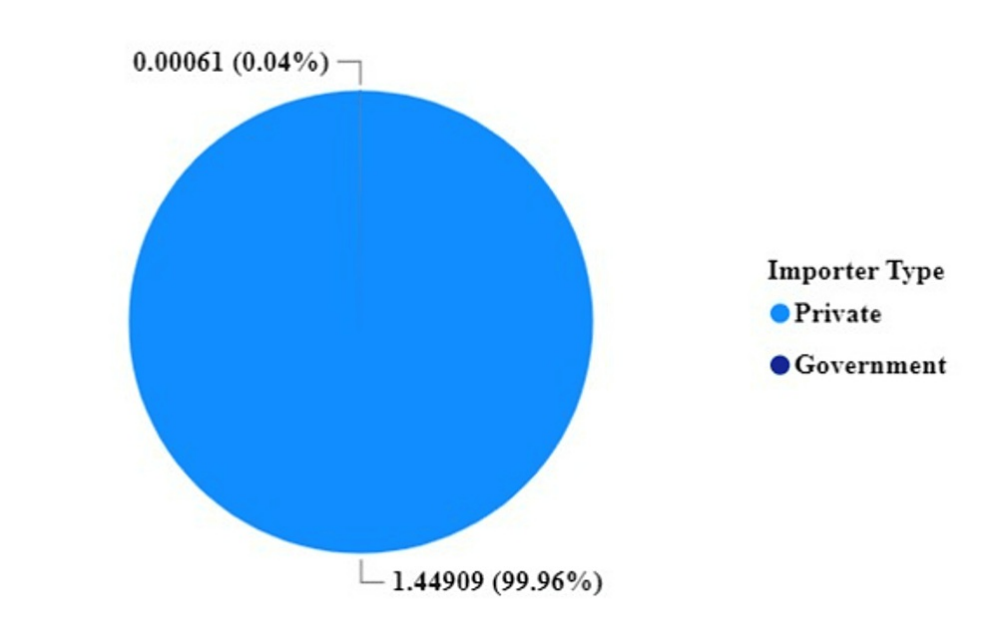
Contribution of the private and public sector in the importation of phosphodiesterase type-5 inhibitors in Tanzania

Compared with private importers, the government imported only 0.04% of all consignments of PDE5Is between 2019 and 2023, whereas private importers imported 99.96% of PDE5Is (Figure 6).

Over the five years studied, the top supplier from India exported PDE5Is, contributing 0.5445 DIDs (37.6%). Eleven suppliers contributed 90% of all the PDE5Is consumed in Tanzania (Table 3).

**Table 3:** Contribution of top suppliers exporting PDE5Is to Tanzania in DID. The total DID over five years was 1.44973 DID: Defined daily doses per 1000 inhabitants per day

Supplier	DID	Cumulative DID	Cumulative %
Supplier 1, India	0.5445	0.5445	37.6
Supplier 2, India	0.2029	0.7474	51.6
Supplier 3, Republic of Yemen	0.13996	0.88736	61.2
Supplier 4, India	0.11847	1.00583	69.4
Supplier 5, India	0.06484	1.07067	73.9
Supplier 6, India	0.05356	1.12423	77.5
Supplier 7, Kenya	0.05067	1.1749	81.0
Supplier 8, India	0.04788	1.22278	84.3
Supplier 9, Egypt	0.03241	1.25519	86.6
Supplier 10, India	0.03133	1.28652	88.7
Supplier 11, India	0.02508	1.3116	90.5

The top local importer made the most outstanding contribution against other local importers, accounting for 0.54528 (37.6%) of all utilizations of PDE5Is. Overall, 11 of the 31 local importers imported 90% of the PDE5Is used in Tanzania during the study period (Table 4).

**Table 4:** Top importers of phosphodiesterase type-5 inhibitors in Tanzania from 2019 to 2023 DID: Defined daily doses per 1000 inhabitants per day

Local Importer	DID	Cumulative DID	Cumulative %
Importer 1	0.54528	0.54528	37.6
Importer 2	0.19744	0.74272	51.2
Importer 3	0.1601	0.90282	62.3
Importer 4	0.0942	0.99702	68.8
Importer 5	0.0758	1.07282	74.0
Importer 6	0.05962	1.13244	78.1
Importer 7	0.04813	1.18057	81.4
Importer 8	0.04376	1.22433	84.5
Importer 9	0.03319	1.25752	86.7
Importer 10	0.03022	1.28774	88.8
Importer 11	0.02662	1.31436	90.7

**Figure 7:**
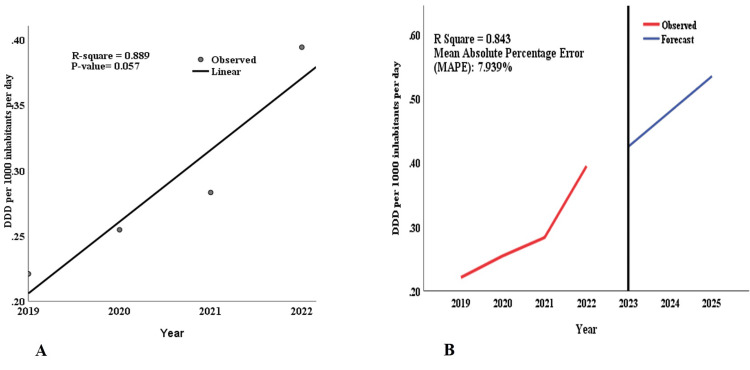
Trends and forecasting of phosphodiesterase type-5 inhibitors (PDE5Is). Utilization from 2019 to 2022, extending to 2025. Panel A reveals an ascending trend in the overall use of PDE5Is, captured through linear curve estimation. Panel B showcases predictions for PDE5Is utilization trends up to 2025, utilizing the Holt linear trend model for a refined projection of future utilization rates DDD: Defined daily dose

Curve-fitting analysis using linear regression on the utilization data in the DID against the year yielded an R-square of 0.889 and a p-value of 0.057. Analysis utilizing the Holt linear trend and ARIMA models predicted increased PDE5Is utilization from 2019 to 2025. The models indicated a strong fit to the data, with an R-square of 0.843 and a mean absolute percentage error (MAPE) of 7.9%. The predicted values increased from 0.220910 DIDs in 2019 to 0.534272 DIDs by 2025. In 2023, the expected utilization was 0.424608 DIDs, compared with the actual observed utilization of 0.29687 DIDs (Figure 7).

## Discussion

An analysis of 587 importation consignments between 2019 and 2023 revealed a discernible trend in the importation and utilization of PDE5Is, with a significant peak in 2022, accounting for 35.1% of imports to mainland Tanzania. This surge is reflected in the utilization data, where 2022 witnessed a DID of 0.39419, indicating a 27.2% share of the five-year cumulative total. The subsequent decline in imports and DID utilization by 2023 suggests fluctuating demand, possibly influenced by external market forces. Notably, sildenafil emerged as the dominant PDE5Is, comprising over three-quarters of total consumption. This preference for sildenafil over tadalafil and vardenafil underscores its central role in managing ED in the country. Other studies have indicated that the propensity for sildenafil over other PDE5Is was 90.6% in Egypt [13]. In the USA, the proportion of prescriptions for the three PDE5Is was 48.7% for sildenafil and 37.8% and 13.5% for tadalafil and vardenafil, respectively [5].

Brands of PDE5Is mostly consumed in Tanzania included Erecto, with a 37.6% share of the total DID over five years, signaling its dominance in the market. Together with 12 other brands-Kamagra, Saheal, Cupid, Penegra, ApcalisSx, Silmelt, Evoke, Novagra, Maxxsat, Silden, Megalis, and Mygra-Erecto constituted 90% of the PDE5Is imported. In contrast, the globally recognized brand Viagra had a minimal share, underscoring significant market differences. This is contrary to initial global reports that indicated the popularity of the Viagra brand [14].

In recent studies, there has been an increase in the use of PDE5Is, particularly sildenafil, mostly known as Viagra or Erecto, and other common names for non-medical or recreational purposes. The majority of users were from younger communities and were not diagnosed with ED, but purchased sildenafil without prescriptions from pharmacies as over-the-counter medications and sometimes through friends without prescriptions. Among the physicians studied in Saudi Arabia, 71.2% used PDE5Is recreationally, 14.4% used them prophylactically, and 14.4% prescribed them [15]. Recreational use of oral PDE5Is may adversely affect the psychological aspects of sexual function [15]. Advocating judicious medication management within healthcare systems and reducing the unnecessary use of PDE5Is are imperative. Moreover, a broader recognition of sexual health and overall well-being should play an integral role for healthcare providers and policymakers in adopting reasonable approaches to ED treatment. Implementation of this approach is critical for mitigating the risks of adverse effects and reducing reliance on pharmacological solutions by addressing the multifaceted causes of ED, including organic, psychogenic, and mixed etiologies [15].

In this study, India emerged as the predominant country supplying PDE5Is to Tanzania, contributing 80% of the PDE5Is utilization from 2019 to 2023, highlighting its central role in the pharmaceutical supply chain. Other key countries included Kenya, Yemen, and Egypt, signifying the diverse sources of PDE5Is entering Tanzania. The role of India’s imports into Tanzania has also been noted [16].

PDE5Is showed peak utilization in November over five years, with high usage rates in May and July. In addition, using the Holt linear trend model, this study confirms an upward trend in PDE5Is utilization by 2025. This increasing utilization pattern mirrors the global escalation of ED cases, necessitating an immediate need for effective ED management that addresses the physical and psychological tolls. This trend is particularly noteworthy against the backdrop of potential medication misuse and associated health risks, as highlighted by anecdotal reports on sildenafil misuse in Tanzania.

Further research extending beyond the years in the present dataset is vital to corroborate these findings and investigate the drivers behind the PDE5Is use surge. Additionally, studies focusing on community behavior and attitudes towards ED treatment outside official data sources, such as TMDA, could offer invaluable insights. Such comprehensive analyses are pertinent to devising effective public health strategies that ensure the rational use of ED medications and tackle the underlying causes and broader societal impacts of the condition.

Clinically, ED is common in older patients and patients with chronic diseases, such as diabetes and hypertension, due to the prolonged use of medications that affect normal blood flow to the peripheral parts [2,17]. Thus, the reported increase in the use of PDE5Is is mostly for recreational purposes, primarily because of their ability to increase blood flow to the penis, thereby helping to maintain erection for an extended period during sex. In Egypt, the majority of those who use PDE5Is purchase them for recreational purposes, mainly pleasure (58.35%) and to increase their time of sexual intercourse (15.6%) [13].

Although sildenafil is approved to be available only upon prescription to men with ED for various reasons, the increased utilization of sildenafil in the country denotes illegal selling and distribution of PDE5Is. A study conducted in the United Kingdom reported that men without ED had a higher tendency to use sildenafil, thereby increasing the abuse of these medicines [18], which was also attributed to the use of recreational drugs such as ketamine and cocaine.

Private importers dominated the market and were responsible for nearly all PDE5I imports, in contrast to minimal public sector involvement. The top supplier significantly contributed to exports to Tanzania's PDE5I supply chain by 37.6%. The government should consider programs to treat EDs and take into account the budget for medicines and the increased number of prescriptions in public hospitals. The non-prescription availability of sildenafil and other PDE5Is could lead to inappropriate use, increased adverse effects, and an increased probability of addiction among users. A study conducted in Addis Ababa, Ethiopia [19] on pharmacist dispensing practices of PDE5Is claimed that most pharmacists knew that PDE5Is are prescription-only medicines but still dispensed them without prescriptions. The motive for this practice was pressure to maximize profit through increased sales of PDE5Is. Private proprietors in the country also own most community pharmacies, intending to return their investments [20] and earn profits. Hence, the pressure to maximize income is high because of the non-prescription selling of PDE5Is, which is still common in Tanzania [20].

A study conducted in Sweden [21] reported that the increase in the use of PDE5Is was also due to the fact that most men have multiple sexual partners. They need to impress their partners and, hence, are involved in the illegal use of these boosters. Multiple sex partners and one-night stands, as often and mostly referred to, are common practices among men in many countries, thereby explaining the rapid increase in import trends and utilization of PDE5Is in society.

Although TMDA and other regulatory authorities have emphasized and kept promulgating educational programs to the public, more cooperation with community pharmacies is needed to deter the illegal supply of these medications [20,22].

The increasing trend in the utilization of PDE5Is implies greater threats and an increased probability of falsified importation of these medicines [22], calling for action from TMDA to curb this problem.

Limitations of the study

The study of PDE5Is utilization in Tanzania, relying on TMDA import data, may not fully encapsulate the dynamics of ED treatment because it focuses solely on official imports, thereby potentially overlooking unofficial sources, re-export activities, and the effects of parallel importation or counterfeit drugs. Furthermore, the significant utilization of traditional or herbal remedies for ED, common in Tanzania but rarely documented formally, remains unaccounted for in this analysis. Limited to Tanzanian data, where other sources of utilization data, such as pharmacy sales data, are unavailable, the applicability of these findings to regions with differing health systems and cultural perspectives on ED is questionable. The retrospective design and quantitative methodologies, including Holt's linear trend and ARIMA models, pose additional challenges as they may not capture nonlinear trends or the comprehensive spectrum of ED management factors such as patient adherence, dose variability, and shifts in national healthcare policies. Given these constraints, the results should be interpreted with caution. Future research should integrate a broader array of data sources and employ quantitative and qualitative methods to gain a more comprehensive understanding of ED treatment practices, thus enhancing the relevance and effectiveness of policies and healthcare strategies in Tanzania and comparable settings.

## Conclusions

In conclusion, our comprehensive analysis of PDE5Is utilization in Tanzania over five years has revealed a significant increase in use through the application of Holt's linear trend model, ARIMA analyses, and the WHO's ATC/DDD methodology for precise utilization quantification. This trend indicates a growing reliance on PDE5Is and presents crucial insights into societal practices.

To address the increasing utilization of PDE5Is, TMDA should monitor the use of PDE5Is and prevent their misuse. Educational campaigns are pivotal to increasing public awareness of ED, including their safe use. This highlights the importance of engaging with professional bodies in the country. Policies promoting the rational use of these medications should be explored to offer healthcare providers clear guidelines for prescription and patient counseling. Further research into alternative treatments is also essential to broaden patient options and potentially reduce dependency on PDE5Is. Finally, fostering global collaboration to exchange knowledge and strategies for managing PDE5Is can help to develop adaptable best practices and policies.
